# Diagnostic Difficulties of Erosive Lichen Planus in a Pediatric Patient

**DOI:** 10.3390/diagnostics15010035

**Published:** 2024-12-27

**Authors:** Carolyn Szwed, Olivia Gudziewski, Marta Sar-Pomian, Malgorzata Olszewska, Lidia Rudnicka, Joanna Czuwara

**Affiliations:** Department of Dermatology, Medical University of Warsaw, 02-006 Warsaw, Poland; carolyn.szwed@wum.edu.pl (C.S.); oliviagudziewski@gmail.com (O.G.); mpomian@gmail.com (M.S.-P.); malgorzataolszewska@yahoo.com (M.O.); lidia.rudnicka@wum.edu.pl (L.R.)

**Keywords:** erosive lichen planus, pediatrics, tongue ulcers, dystrophic nails, esophagus, LC-OCT

## Abstract

**Background:** Lichen planus (LP) is a chronic inflammatory disease that can present with significant morbidity, particularly in children. Erosive lichen planus (ELP), its rare destructive subtype, can be particularly difficult to diagnose and manage. We present a rare pediatric case of ELP with multisite involvement and discuss the differential diagnosis. **Case **Presentation**:** A 12-year-old boy presented with painful erosions and ulcers on the lateral tongue and dystrophic nails. His six-year history of tongue and nail lesions prompted several comprehensive examinations. Laboratory tests did not reveal any abnormalities. Histopathological examination of the tongue lesions was representative of ELP. Line-field confocal optical coherence tomography (LC-OCT) examination of the tongue lesions showed features that strongly correlated with histopathology. The patient was later hospitalized due to dysphagia and esophageal food impaction, during which esophageal ELP was confirmed. The patient was initially managed with topical corticosteroids. He was later started on systemic therapy in the form of methotrexate and low-dose naltrexone to address his symptoms and disease presentation. **Conclusions:** This case highlights the complexities of diagnosis and management of ELP in pediatric patients. A multidisciplinary approach and regular follow-up are necessary to manage symptoms, prevent complications, and improve quality of life.

## 1. Introduction

Erosive lichen planus (ELP) is a chronic inflammatory disease that primarily affects the mucous membranes, particularly within the oral cavity [1]. It is characterized by painful erosions and ulcers that heal with scarring. ELP can present with a white lacy pattern known as Wickham striae, which can be helpful for clinical differentiation and diagnosis [2]. The exact etiology and pathogenesis remain unclear, although a T-cell-mediated immune response is believed to lead to the destruction of basal keratinocytes [3]. Several exogenous factors, such as allergens, drugs, viruses, and stress, have also been implicated in the development of the disease [4]. Patients often experience discomfort and difficulty with eating and swallowing, leading to a significant impact on quality of life [5].

Diagnosis typically involves clinical evaluation and a biopsy to exclude other diagnoses. Management focuses on relieving symptoms and preventing complications. Early diagnosis and treatment of ELP are crucial, particularly in children. We present a rare pediatric case of ELP with multisite involvement and discuss the differential diagnosis.

## 2. Case Presentation

**Figure 1:**
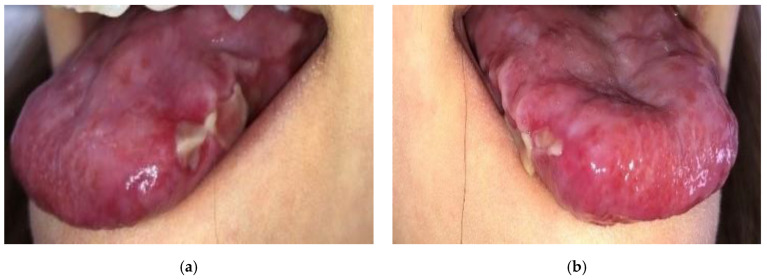
Deformed tongue. (**a**) Papillary atrophy and erosions and ulcers covered with fibrin on the left lateral aspect of the tongue; (**b**) diffuse ulcers on the right lateral aspect of the tongue.

**Figure 2:**
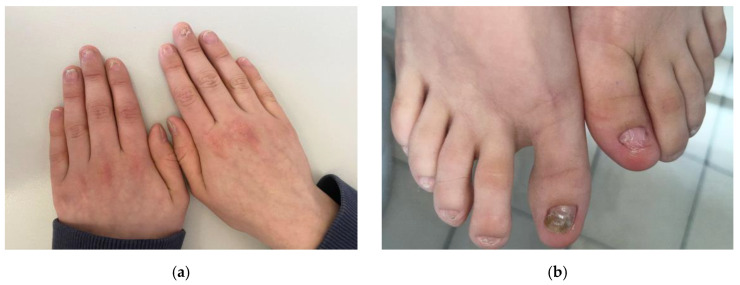
Dystrophic nails. (**a**) Brittleness, ridging, splitting, and dorsal pterygium of the fingernails; (**b**) brittleness and ridging of the toenails with the great toenails most affected.

A 12-year-old boy presented to our dermatological department with a primary complaint of painful, persistent erosions and ulcers on the lateral tongue (Figure 1). Papillary atrophy of the tongue with its deformity was also noted. He also exhibited dystrophic nails, characterized by ridging and splitting (Figure 2). No cutaneous lesions were observed. His family history was not contributory. The boy’s height and weight were at the 25th percentile for his age. He was previously suspected of having bacterial and fungal infections as well as metabolic disturbances and nutritional and vitamin deficiencies. However, oral bacterial and fungal cultures, metabolic panels, and amino acid analysis revealed no abnormalities. Serum HBsAg, anti-HCV antibodies, and antinuclear antibodies (ANA) were negative. His six-year history of tongue and nail lesions prompted several comprehensive examinations, including laboratory tests, tongue biopsies, and real-time non-invasive imaging with line-field confocal optical coherence tomography (LC-OCT).

**Figure 3:**
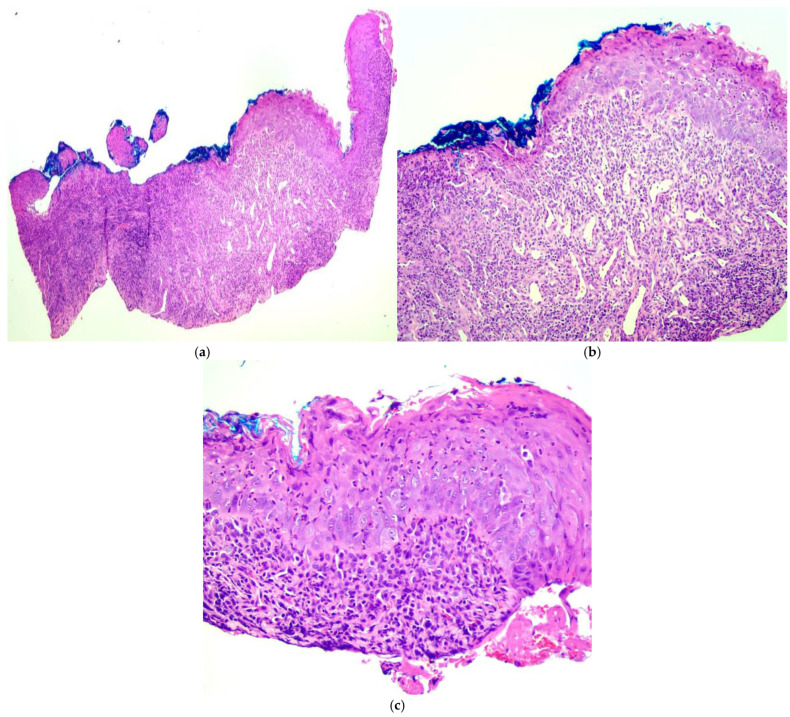
Histopathological examination of the tongue lesions (hematoxylin & eosin stain). (**a**) Stratified squamous epithelium eroded in the center with inflammation and granulation tissue in the lamina propria (40×); (**b**) atrophic and flattened epithelium with basal layer degeneration, reactive atypia, and keratinocyte dysmaturation with a prominent accompanying exocytosis of lymphocytes and neutrophils (100×); (**c**) apoptotic and dyskeratotic keratinocytes in the epithelium with squamatization of the basal layer, which is obscured by a dense band-like infiltrate composed primarily of lymphocytes and plasma cells (200×).

Laboratory tests were all within normal range. Histopathological examination of the tongue lesions revealed a stratified squamous epithelium with features of epithelial atrophy and flattening and superficial lymphocytic exocytosis. Additionally, numerous apoptotic and dyskeratotic keratinocytes were present. Deeper sections showed a dense band-like inflammatory infiltrate within the superficial lamina propria, composed of lymphocytes, plasma cells, and neutrophils, as well as the formation of granulation tissue underlying the ulcers. The granulation tissue exhibited dilated blood vessels and endothelial cell stimulation. Prominent basal layer degeneration and squamatization were also present in the adjacent epithelium. Ultimately, histopathological examination demonstrated lichenoid inflammation consistent with a diagnosis of LP, which, in combination with ulcer formation, corresponded to ELP (Figure 3). Autoimmune bullous diseases were excluded in the patient due to the negative detection of IgG, IgA, IgM, and C3 deposits via direct immunofluorescence testing on the tongue biopsy sample.

**Figure 4:**
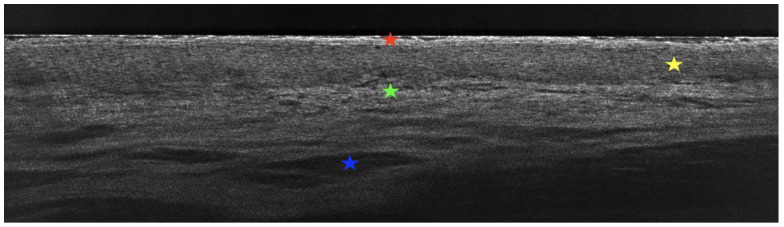
Line-field confocal optical coherence tomography (LC-OCT) of the tongue lesions in the vertical section. Hyperkeratosis (red star) and an atrophic and flattened epithelium (yellow star). Bright, round inflammatory cells, representative of dense lichenoid inflammatory infiltrate (green star), and numerous dilated blood vessels within the lamina propria (blue star).

**Figure 5:**
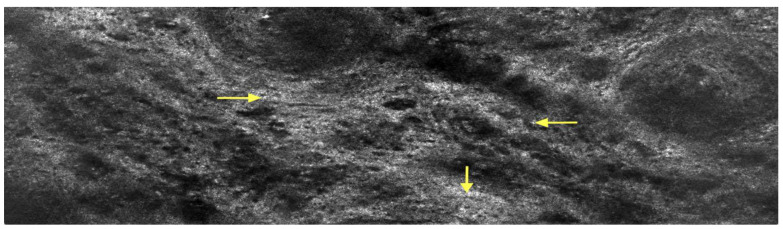
Line-field confocal optical coherence tomography (LC-OCT) of the tongue lesions in the horizontal section. The dense lichenoid inflammatory infiltrate seen on the vertical section is highlighted (yellow arrows) along with an absence of evident lamina propria papillae.

LC-OCT examination of the tongue lesions in the vertical section showed hyperkeratosis and an atrophic and flattened epithelium. Additionally, bright, round inflammatory cells representative of the dense lichenoid inflammatory infiltrate seen on histopathological examination were visible. Numerous dilated blood vessels within the lamina propria were also observed (Figure 4). LC-OCT examination of the tongue lesions in the horizontal section further highlighted the dense lichenoid inflammatory infiltrate seen on the vertical section as well as the absence of evident lamina propria papillae. The LC-OCT features strongly correlated with the histopathological features, confirming active ELP (Figure 5).

After his initial dermatological evaluation and diagnosis of ELP of the tongue, the patient was hospitalized in another center due to dysphagia and esophageal food impaction. The impacted food bolus was visualized and removed endoscopically. Strictures were noted in the proximal one-third of the esophagus, in addition to many areas of mucosal denudation and scarring. Several biopsies were performed, and the microscopic features confirmed ELP of the esophagus.

Initial management included topical therapies, such as fluocinolone acetonide gel on the affected areas of the tongue, which provided only partial relief. Gastroenterologists also recommended budesonide to swallow twice a day. After three months of such treatment, the control endoscopy revealed partially relieved esophageal stenosis. A decision to initiate systemic immunosuppressive therapy was made due to the persistence of symptoms and the involvement of the esophagus. The patient was started on methotrexate (10 mg/week), folic acid supplementation (15 mg/week), and low-dose oral naltrexone (4.5 mg/day). Regular follow-up was planned to monitor his symptoms and treatment response.

## 3. Discussion

Erosive lichen planus (ELP) is a severe variant of LP, primarily affecting the oral and/or genital mucous membranes but also the palmar and/or plantar surfaces. While it can occur in individuals of all ages, it rarely affects children [6]. The disease has a slight female predominance and is often associated with other autoimmune conditions. Oral mucosal involvement is a hallmark of ELP. The entity typically presents with painful erosions and ulcers involving the buccal mucosa, gingiva, and, as in the case of our patient, tongue [7]. Although ELP most commonly affects the oral cavity, it can involve other sites such as the genitals, esophagus, conjunctiva, larynx, anus, and urinary bladder [8]. Regardless of initial symptoms, a thorough clinical examination should be performed due to the tendency of patients to develop disease in more than one site [9].

Our patient was confirmed to have esophageal involvement in ELP during hospitalization in another center. Esophageal involvement in LP is seldom described. It is believed to be an underdiagnosed condition due to its nonspecific clinical presentation [10]. Patients, most often middle-aged women, usually present with dysphagia, odynophagia, weight loss, and food impaction. It is additionally worth noting that malignant transformation of esophageal LP is frequent [11]. Esophageal involvement should be investigated in patients with confirmed LP of other sites and esophageal-related symptoms. Our patient presented with classic symptoms and is a particularly rare example of pediatric esophageal ELP.

The diagnosis of ELP is typically established based on clinical presentation. A biopsy can be helpful to confirm the diagnosis and exclude other entities. An overview of the differential diagnosis of ELP with an emphasis on oral mucosal involvement is provided in Table 1. All possible differential diagnoses in the pediatric population, such as aphthous ulcers, Behçet’s disease, Crohn’s disease, and morsicatio mucosae oris, among others, are listed in the order of probability.

Histopathologically, mucosal LP is characterized by the presence of hyperkeratosis, wedge-shaped hypergranulosis, acanthosis, basal layer degeneration, saw-toothing of the rete ridges, and a dense band-like inflammatory infiltrate along the basal layer composed primarily of lymphocytes and plasma cells [12,13]. These features may be difficult to observe in ELP due to the presence of erosions and ulcers and the subsequent loss of epithelium with the presence of neutrophils, which are not typical cells for LP but are typical for damaged, necrotic, and regenerating tissue.

LC-OCT, a novel non-invasive imaging technique, has been mainly used for the skin [14], but it also has potential for assessing the mucous membranes. In the case of the mucosa, LC-OCT allows real-time, in vivo evaluation of the epithelium and the underlying lamina propria. To the best of our knowledge, this is the first report describing the use of LC-OCT for evaluating the tongue, in particular ELP of the tongue. LC-OCT examination has previously been described for cutaneous LP. In the case of cutaneous LP, authors noted the presence of thickened stratum corneum, thickened epidermis, and a poorly defined dermal–epidermal junction in the vertical section. In the horizontal section, they observed the absence of evident dermal papillae and the presence of small, bright inflammatory cells [15]. In our case of ELP of the tongue, we noted several similar features. This new diagnostic technique requires assessment of a larger group of patients, but it appears to be a valuable tool for evaluating disease severity as well as treatment response. We plan to perform additional LC-OCT examinations during the patient’s follow-up.

Treatment of ELP remains a challenge due to frequent relapses [16]. Knowledge of therapeutic options is limited, and there is a current lack of recommendations regarding the management of pediatric ELP. The available literature is insufficient and based primarily on case reports or series. Currently, topical corticosteroids remain the mainstay of treatment. Also, topical calcineurin inhibitors are frequently employed. Occasionally, short courses of oral corticosteroids may be used for symptomatic flares. In refractory cases, retinoids, dapsone, hydroxychloroquine, and other immunosuppressive agents, including methotrexate, azathioprine, and cyclosporine, may be used [17]. Low-dose naltrexone can also be used in ELP. This treatment modality has an anti-inflammatory effect, and patients have demonstrated clinical improvement in other forms of LP, particularly nail LP [18]. Success with efalizumab, a monoclonal anti-CD11a antibody [19], and tofacitinib, a Janus kinase inhibitor, has also been reported [20].

We chose to initiate systemic treatment in the form of methotrexate and low-dose naltrexone due to the persistence of symptoms despite the use of topical corticosteroids and the patient’s esophageal involvement. We selected methotrexate based on the good clinical experience that our center has with its use in LP. Methotrexate is well tolerated in the pediatric population, allows tailored dosing, and is considered safe for long-term use.

Onychodystrophy is considered a part of palmoplantar ELP. In our patient, dystrophic features typical for nail LP were observed without acral skin involvement. The decision to perform a nail biopsy was abandoned in this 12-year-old boy due to potential unnecessary trauma and his characteristic nail features.

ELP usually leads to the development of systemic symptoms and scarring. It can also lead to several complications, such as secondary infections and malignant transformation [21]. Therefore, regular follow-up, at least biannually, is recommended [22].

## 4. Conclusions

Erosive lichen planus (ELP) is a debilitating disease that can involve multiple sites. Patients tend to respond poorly to topical treatment, and knowledge of effective therapeutics is limited, particularly in pediatrics. A multidisciplinary approach is necessary in order to provide symptomatic relief and improve quality of life. This subtype of LP is rare, particularly in children, but it should be considered in the context of extensive mucosal lesions. The skin may be spared. Additional symptoms, such as dysphagia, may indicate esophageal involvement and should prompt biopsies to confirm. Ultimately, regular follow-up with ELP patients is crucial for improving our understanding and therapeutic approach to this infrequent childhood condition.

## Figures and Tables

**Table 1:** Differential diagnosis of erosive lichen planus (ELP) with an emphasis on oral mucosal involvement based on clinical and histopathological findings (GI: gastrointestinal; ANA: antinuclear antibodies; DIF: direct immunofluorescence; IIF: indirect immunofluorescence; HPV: human papillomavirus).

Disease	Differentiating Features
Aphthous ulcers	Recurrent; can appear alone or secondary to many distinct diseases. Tend to be small and shallow with a tendency to resolve within a few days. Histopathology: nonspecific with a mixed cellular infiltrate due to superficial secondary bacterial infection.
Behçet’s disease	Chronic and relapsing course. Recurrent oral aphthous ulcers, genital ulcers, and uveitis. Cutaneous lesions include acneiform papules, pustules, and furuncles. Pathergy. Histopathology: neutrophilic vasculitis.
Crohn’s disease	Chronic course. Involvement of other areas of the GI tract, +/- associated GI symptoms. Histopathology: non-caseating, ill-defined granulomas surrounded by lymphocytes.
Morsicatio mucosae oris	Chronic course. Patient follow-up is crucial. Histopathology: acanthotic or hyperplastic epithelium with hyperparakeratosis, top clefts lined by bacteria, and little inflammation.
Oral lupus erythematosus	Detection of ANA can be helpful. Systemic involvement with the exception of chronic discoid lupus erythematosus. Histopathology: lichenoid mucositis with vacuolar degeneration with a deep perivascular inflammatory infiltrate composed of lymphocytes and plasma cells; thickening of the basement membrane can be helpful.
Oropharyngeal candidiasis	White lesions that can be easily removed. Histopathology: branching pseudohyphae in the keratin layer and variable inflammation within the epithelium with neutrophils.
Pemphigus vulgaris	Erosions and painful flaccid blisters. DIF: detection of intercellular antibodies in vivo in the subclinically affected epithelium. IIF: detection of circulating antibodies against desmoglein 1 and desmoglein 3. Histopathology: suprabasal acantholysis, intact basal layer, intraepithelial eosinophils, and occasional neutrophils.
Paraneoplastic pemphigus	Erosions and painful flaccid blisters. DIF: detection of intercellular antibodies in vivo in the subclinically affected epithelium. IIF: detection of circulating antibodies against desmoglein 1, desmoglein 3, envoplakin, and periplakin. Histopathology: variable with features of pemphigus vulgaris, lichenoid and vacuolar inflammation, and rarely eosinophils.
Oral leukoplakia	Tobacco history. Premalignant condition. White plaque that cannot be easily removed. Histopathology: epithelial hyperplasia with dysplastic suprabasal keratinocytes and loss of proper stratification and polarity; chronic inflammatory infiltrate may be present.
Proliferative verrucous leukoplakia	Aggressive premalignant condition. White hyperkeratotic verruciform lesions typically found on the gingiva. Histopathology: can mimic oral lichen planus but has a verrucoid epithelial architecture and basal layer degeneration is typically absent.
Oral squamous cell carcinoma	Malignancy related to oncogenic HPV types, tobacco, or other underlying risk factors. Histopathology: hyperplastic epithelium with full thickness atypia, endophytic growth and invasion, dyskeratosis and keratin pearls, intercellular bridges, cellular and nuclear pleomorphism, and mitotic activity.

## Data Availability

Further inquiries should be directed to the corresponding author.
